# Is low dose of liposomal amphotericin B effective in management of acute invasive fungal rhinosinusitis? Our conclusions from Al-Mowassat University Hospital, Syria: a prospective observational study

**DOI:** 10.1186/s12879-023-08177-0

**Published:** 2023-03-31

**Authors:** Muhammad Nour Alabdullah, Abdulmajeed Yousfan

**Affiliations:** Otorhinolaryngology Department, Al-Mowassat University Hospital, Faculty of Medicine, Damascus University, Damascus, Syrian Arab Republic

**Keywords:** AIFRS, Invasive fungal sinusitis, Amphotericin B, Diabetes mellitus, COVID-19, Glucocorticoid, Syria

## Abstract

**Background:**

Acute invasive fungal rhinosinusitis (AIFRS) is a fatal infection associated with high morbidity and mortality. Although it is a rare disease, upsurge of AIFRS was noticed during the second wave of COVID-19 disease. Early diagnosis and management is the cornerstone for good outcomes. However, management of AIFRS is challengeable especially in developing countries due to limited resources and high prices of antifungal agents. No previous studies have been conducted to evaluate the outcomes of management of AIFRS in Syria. The purpose of this study is to report the results of management of AIFRS with low doses of liposomal amphotericin B in our tertiary hospital in Syria.

**Methods:**

The outcomes of management of AIFRS cases were followed through a prospective observational study between January 2021 and July 2022. The required medical data were collected for each individual. Three-month mortality rate was studied. SPSS v.26 was used to perform the statistical analysis. Pearson Chi-square test was used to study the associations between different variables and mortality. Survival curves were plotted by the Kaplan–Meier to compare the survival probability. Log Rank (Mantel-Cox) test and Cox regression were conducted to evaluate the factors affecting survival within the follow up period.

**Results:**

Of 70 cases, 36 (51.4%) were males and 34 (48.6%) were females. The mean age of patients was 52.5 years old. The most common underlying risk factor was diabetes mellitus (84.3%). The used dose of liposomal amphotericin B ranged between 2–3 mg/kg per day. The overall 3-month mortality rate was 35.7%. Significant association was found between survival and the following variables: Age, orbital involvement, stage, and comorbidity.

**Conclusion:**

The overall mortality rate was close to other studies. However, survival rate was worse than comparable studies in selected cases of AIFRS (older ages, involved orbits, advanced stages, and chronic immunodeficiency). Therefore, low doses of liposomal amphotericin B could be less effective in such cases and high doses are recommended.

## Background

Acute invasive fungal rhinosinusitis (AIFRS) is an opportunistic infection occurs when ubiquitous fungi are being inhaled into the nasal cavity and invade sinonasal mucosa, submucosa, vasculature or bone, leading to tissue ischemia and necrosis [1]. It is one of the most devastating diseases of nasal cavity and paranasal sinuses, characterized by fungal hyphae invading tissue structures within 4 weeks or less, leading to high morbidity and mortality in immunocompromised hosts [2, 3]. AIFRS is a fatal infection that has the ability to develop and spread quickly into the orbital and intracranial structures [4]. It is rare, accounting for about 2% of immunocompromised patients [5]. The most common pathogenic fungi are Mucor and Aspergillus species. However, several species have been reported to be the causative organisms including Candida, Alternaria, Fusarium and Scedosporium [1, 6]. Several types of mucormycosis have been described including pulmonary, gastrointestinal, cutaneous, and disseminated mucormycosis [7]. However, according to a large study, rhino-orbito-cerebral mucormycosis (ROCM) is the most common type of the disease [8]. In the twentieth century, diabetes was the major risk factor for AIFRS. In the last two decades, however, underlying malignancies appeared to be another risk factor due to management by chemotherapy or cancer immunotherapy. Hematopoietic stem-cell and solid organ transplantations represent another risk factors [6]. Several other risk factors for AIFRS have been reported including glucocorticoids, long-term use of antibiotics, acute granulocytopenia, severe burns, long-term use of immunosuppressants after organ transplantation, and AIDS [1, 6]. Recently, the COVID-19 infection was considered one of the most important risk factors after a significant number of COVID-19 patients have been infected with AIFRS with high morbidity and mortality rates [9, 10]. The outcomes of management of AIFRS are heterogeneous but, however, unfavorable. The previous studies reported a variable mortality rate, ranging from 33 to 80% [1, 4, 11]. Early diagnosis and management are the cornerstone for good outcomes. However, this could be challengeable due to nonspecific characteristics. The diagnosis is based on a combination of clinical manifestations, endoscopic signs, radiologic findings, and histopathological examination [4, 12]. The presenting symptoms of AIFRS are vague and unspecific including facial swelling, nasal congestion, rhinorrhea, fever, headache, and facial pain. The last two symptoms are the most common [11, 13, 14]. Alarming symptoms and signs such as facial swelling, numbness or pain, visual changes, blacken mucosa, exophthalmos, and orbital or intracranial invasion should place the AIFRS at the top of the differential diagnoses particularly in immunocompromised patients [15]. Computed tomography (CT) and magnetic resonance imaging (MRI) are useful tools for the diagnosis, treatment planning, and follow up. Despite the findings of CT are nonspecific, it can help to detect early infection by revealing thickening of the sinonasal mucosa. However, the modality of choice for intraorbital or intracranial spreading of disease is MRI [16–18]. Treatment of AIFRS is directed to stop the progression of the disease. However, the optimal regimen for effective management has not yet been defined. The following steps resemble the cornerstone of management: early diagnosis, immediate start with antifungal therapy, reverse the immunocompromised state, and aggressive surgical debridement [19, 20]. An upsurge of number of AIFRS cases in Syria was noticed during COVID-19 pandemic especially after the second wave of disease. The number increased from about 1–3 cases per year to about 100 cases during the 2021. Ensuring antifungal agents was unaffordable by many patients due to its high prices. Therefore, AIFRS cases were treated with low doses of amphotericin B by medical centers of Syria. The aim of this paper is to review our experience dealing with AIFRS, and to report the outcomes of management in our hospital, which is the largest tertiary health care center in Syria.

## Methods and materials

### Study design

A prospective observational study was conducted to follow the results of management of the patients who were diagnosed with AIFRS during the period between January 2021 and July 2022 in the department of otolaryngology, head and neck surgery of Al-Mowassat University Hospital which is a tertiary teaching hospital in Syria. This study is a part of an ongoing Master's Research Project and approved by the Research Ethics Committee, Faculty of Medicine, Damascus University, and was conducted according to the declaration of Helsinki and its later amendment. Follow up was performed weekly until the end of the treatment. Short-term survival was assessed 3 months after the end of the treatment.

### Study participants

Patients with diagnosis of proven AIFRS, depending on The European Confederation of Medical Mycology (ECMM) and the Mycoses Study Group Education and Research Consortium (MSG ERC) criteria [21], were included in the study. Exclusion criteria included that the patient: (1) has incomplete medical records, (2) did not complete the treatment, (3) had other types of mucormycosis (pulmonary, gastrointestinal, cutaneous, disseminated mucormycosis). Each patient's medical and personal history has been reviewed with a focus on aspects including socio-demographic characteristics (age, gender), clinical features, comorbidities (such as diabetes mellitus, immunodeficiency diseases, previous infection with COVID-19 disease confirmed by PCR or CT chest), radiological findings, ophthalmological findings, previous medications received (such as systemic steroids, immunosuppressant drugs), the interval between the onset of symptoms and the commencement of treatment, histopathological examinations, direct microscopic examination and/or fungal culture, management strategies and 3-month follow-up. Rhinoscopy (for visual assessment of the nasal cavity using a rigid or flexible fiberoptic endoscope) and radiological assessment of nasal cavity, paranasal sinuses, orbit and central nervous system (CNS) using CT and/or MRI were undertaken to define the extent of disease. The patients were classified into four stages according to extent of the disease (Table 1).

**Table 1:** The proposed staging system of AIFRS disease

Stage of AIFRS	Extent of disease
Stage 1	Involvement of the nasal mucosa
Stage 2	Involvement of the paranasal mucosa
Stage 3	Involvement of the orbit
Stage 4	Involvement of the CNS

*CNS* central nervous system

### Data analysis

All data were analyzed using IBM SPSS Statistics version 26. Continuous variables were summarized as means and standard deviations (SDs). Categorical variables were reported as frequencies and percentages. Pearson Chi-square univariate analysis was used to make the necessary comparisons and identify the correlation between different categorical variables. Survival analysis was plotted by the Kaplan–Meier method, and Log Rank (Mantel-Cox) test was applied to compare the survival curves of several variables (age, gender, staging, orbital involvement, and comorbidity). Univariate and multivariate Cox regression test were performed to identify the prognostic factors associated with overall survival. A *p* value of < 0.05 was considered statistically significant.

## Results

**Fig. 1:**
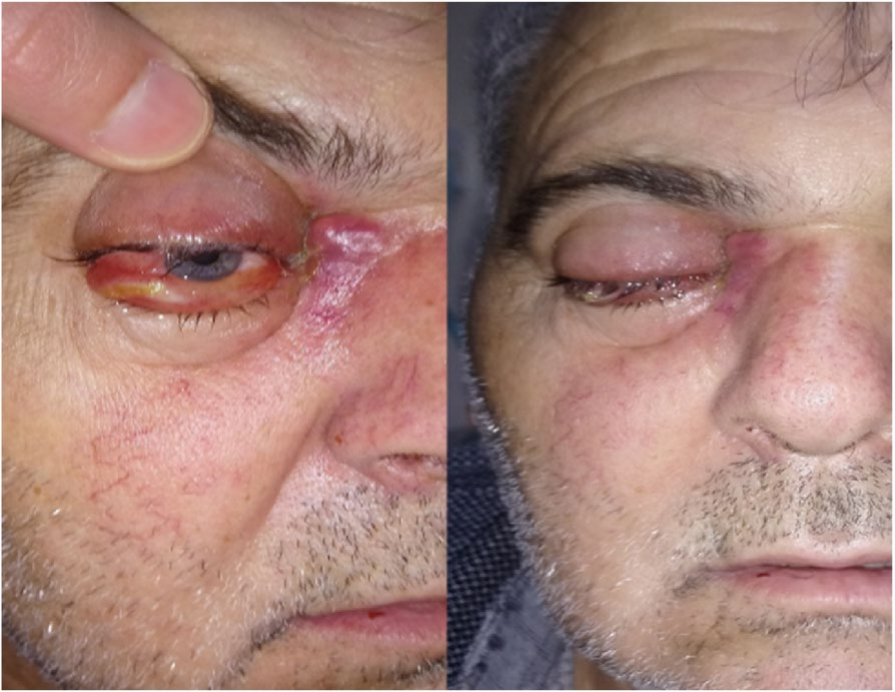
Clinical picture of AIFRS patient shows ptosis, chemosis, edema and discoloration of the eyelid and periorbital region

Eighty-two patients with AIFRS were identified. Of them, 70 patients were included, while 12 patients were excluded due to incomplete medical records (8 cases), incomplete treatment (3 cases), or refusal the participation (1 case). The mean age of included patients was 52.5 years (SD 14.2; range 24–89). Of the patients, 36 (51.4%) were male and 34 (48.6%) were female. Regarding to underlying comorbidities, diabetes mellitus (DM) was found in 59 cases (84.3%), which was ongoing in 33 patients (47.1%) and recently discovered (denovo) in 26 patients (37.1%). Of the 33 patients of ongoing DM, 13 patients (18.6%) had previous history of COVID-19 infection with systemic steroid therapy, while 17 patients (24.3%) from denovo DM group had previous history of COVID-19 infection with systemic steroid therapy. Four patients (5.7%) had chronic kidney disease (CKD). Three patients (4.3%) had acute myeloid leukemia (AML). One patient (1.4%) was kidney transplant recipient and placed on immunosuppressive therapy. COVID-19 infection with systemic steroid therapy were the only apparent risk factors for 3 patients (4.3%). The most common initial symptoms were facial pain and swelling (49 cases, 70%) followed by headache (31 cases, 44.3%). Other symptoms included facial numbness (41 cases, 58.6%), nasal congestion (46 cases, 65.7%), visual deterioration (21 cases, 30%), eyelid drooping (22 cases, 31.4%), restricted eye movements (12 cases, 17.1%) (Fig. 1). CT and/or MRI were performed for all patients. The infection was limited to nasal mucosa (stage 1) in 7 cases (10%), paranasal sinuses (stage 2) in 29 cases (41.4%), the orbit (stage 3) in 25 cases (35.7%), and spreading to CNS (stage 4) in 9 cases (12.9%). Orbit involving disease was found in 34 patients (48.6%). AIFRS affected the right side in 40 cases (57.1%), the left side in 28 cases (40%), and both sides in 2 cases (2.9%). The interval between the onset of symptoms and commencement of treatment ranged from 4 to 21 days (mean 9.2; SD 4.3) for patients with orbit sparing disease, and 2 to 13 days (mean 6.5; SD 2.4) for patients with orbit involving disease. A combination of medical and surgical intervention was applied in management of 66 cases (94.3%). The remaining 4 patients (5.7%) received medical treatment only. All of the 66 patients (100%), who were managed surgically, underwent transnasal endoscopic sinus surgery (TESS). In addition to TESS, external approach was used in 4 patients (6.1%), and orbital exenteration was performed in 4 patients (6.1%). The number of surgical interventions ranged from 1 to 3 (mean 2.1; SD 0.6). Ten patients (15.2%) needed just one surgical debridement, 38 patients (57.6%) needed 2 surgical debridements, and the remaining 18 patients (27.3%) needed 3 surgical debridements. Surgical samples were sent for direct microscopic examination and/or fungal culture. Causative fungi were Mucor (50 cases; 71.4%), Aspergillus (4 cases, 5.7%), co-infection with Mucor and Aspergillus (3 cases; 4.3%). However, no data were available for the remaining 13 cases (18.6%). All the 70 patients received intravenous liposomal amphotericin B. The mean dosage per day was 170.6 mg (SD 28.3; range 150–250). In addition to liposomal amphotericin B, posaconazole has been added for management of aspergillosis and co-infection cases (300 mg twice a day on day 1 and once a day from day 2). The period of treatment ranged between 4–12 weeks (mean 8.6; SD 2.2). All patients were discharged home with a prescription of oral posaconazole maintenance therapy (300 mg twice a day on day 1 and once a day from day 2) for 1 month. Ten cases (14.3%) recurred and underwent repeat debridement with intravenous liposomal amphotericin B. After follow-up of patients within 1–3 months, the total number of deaths was 25 cases (35.7%). Characteristics of patients with AIFRS are summarized in Table 2.

**Table 2:** Characteristics of patients with AIFRS (*n* = 70)

	No. (%)
Age (mean ± SD, range)	52.5 ± 14.2, 24–89 years
Gender	
Male	36 (51.4)
Female	34 (48.6)
Comorbidities/Risk factors	
Ongoing DM	33 (47.1)
Denovo DM	26 (37.1)
CKD	4 (5.7)
AML	3 (4.3)
Kidney transplantation	1 (1.4)
COVID-19 + Systemic steroid	3 (4.3)
Presenting symptoms	
Facial pain and swelling	49 (70)
Headache	31 (44.3)
Facial numbness	41 (58.6)
Nasal congestion	46 (65.7)
Visual deterioration	21 (30)
Eyelid drooping	22 (31.4)
Restricted eye movements	12 (17.1)
Stage	
Stage 1	7 (10)
Stage 2	29 (41.4)
Stage 3	25 (35.7)
Stage 4	9 (12.9)
Orbital involvement	
Orbit involving disease	34 (48.6)
Orbit sparing disease	36 (51.4)
Affected side	
Right	40 (57.1)
Left	28 (40)
both	2 (2.9)
Organism	
Mucor	50 (71.4)
Aspergillus	4 (5.7)
Both	3 (4.3)
No data	13 (18.6)
Outcome	
Died	25 (35.7)
Survived	45 (64.3)

*DM* diabetes mellitus, *CKD* chronic kidney disease, *AML* acute myeloid leukemia

### Correlations between survival and patients characteristics

According to Kaplan–Meier survival analysis and Log Rank (Mantel-Cox) test, the overall short-term survival rate was 64.3%. There was no significant association between the survival of patients and gender (*P* = 0.7). Significant associations were found between age (*P* = 0.0004), orbital involvement (*P* ≤ 0.0001), stage (*P* ≤ 0.0001), comorbidity (*P* = 0.0002) and survival of patient. Using the Chi-square univariate analysis (Table 3), the results revealed that no significant correlation was found between mortality and the following variables: gender (*P* = 0.7), comorbidity (*P* = 0.07), causative fungi (*P* = 0.2). A significant association was found between the age groups and mortality, where mortality was significantly higher in patients over 65 years of age (81.8%, P-value = 0.0001). Orbital involvement was significantly correlated to mortality, where orbit involving cases had higher mortality rate compared to orbit sparing cases (61.8 vs. 11.1%, *P*-value < 0.0001). The stage of disease was significant associated with mortality rate (< 0.0001), where mortality rate was clearly higher in stage 3 and 4 (56%, 77.8%, respectively). Regarding to comorbidity, no significant correlation was found between different comorbidities and mortality (*P-*value = 0.07). The interval between the onset of symptoms and commencement of treatment was associated with mortality rate (*P*-value < 0.0001). Univariate Cox regression analysis revealed that the age, stage, orbital involvement, and comorbidity had a significant prognostic effect on survival function (Table 4). On the other hand, multivariate Cox regression analysis showed that the age, stage, and comorbidity had a significant prognostic effect on survival function (*p* < 0.05) (Table 5).

**Table 3:** Correlation between survival and participants’ characteristics

	died	survived	Pearson Chi-square value	*P*-value
Age				
≤ 24 (*n* = 1)	0.00	1 (100%)	21.059	0.0001
25–44 (*n* = 18)	0.00	18 (100%)		
45–65 (*n* = 40)	16 (40%)	24 (60%)		
≥ 65 (*n* = 11)	9 (81.8%)	2 (18.2%)		
Gender				
Male (*n* = 36)	12 (33.3%)	24 (66.7%)	0.183	0.6
Female (*n* = 34)	13 (38.2%)	21 (61.8%)		
Orbital involvement				
Involved (*n* = 34)	21 (61.8%)	13 (38.2%)	19.541	< 0.0001
Not involved (*n* = 36)	4 (11.1%)	32 (88.9%)		
Stage				
Stage 1 (*n* = 7)	0.00	7 (100%)	21.375	< 0.0001
Stage 2 (*n* = 29)	4 (13.8%)	25 (86.2%)		
Stage 3 (*n* = 25)	14 (56%)	11 (44%)		
Stage 4 (*n* = 9)	7 (77.8%)	2 (22.2%)		
Comorbidity				
Ongoing DM (*n* = 33)	13 (39.4%)	20 (60.6%)	10.136	0.07
Denovo DM (*n* = 26)	7 (26.9%)	19 (73.1%)		
CKD (*n* = 4)	1 (25%)	3 (75%)		
AML (*n* = 3)	3 (100%)	0.00		
Kidney transplantation (*n* = 1)	1 (100%)	0.00		
COVID-19 + Systemic steroid (*n* = 3)	0.00	3 (100%)		
Organism				
Mucor (*n* = 50)	17 (34%)	33 (66%)	4.462	0.2
Aspergillus (*n* = 4)	3 (75%)	1 (25%)		
Both (*n* = 3)	0.00	3 (100%)		
No data (*n* = 13)	5 (38.5%)	8 (61.5%)		
Time of treatment^a^				
≤ 7 days (*n* = 39)	23 (59%)	16 (41%)	20.813	< 0.0001
8–14 days (*n* = 28)	2 (7.1%)	26 (92.9%)		
15–21 days (*n* = 3)	0.00	3 (100%)		

*DM* diabetes mellitus, *CKD* chronic kidney disease, *AML* acute myeloid leukemia

^a^The interval between the onset of symptoms and commencement of treatment

**Table 4:** Univariate Cox regression analysis of the prognostic factors associated with survival

Variable	HR (95% CI)	*P*-value
Age	1.135 (1.085–1.187)	< 0.0001
Stage	3.425 (2.000–5.866)	< 0.0001
Number of surgical interventions	1.187 (0.729–1.933)	0.490
Gender		
Male	Reference 1.173 (0.532–2.588)	0.692
Female		
Orbital involvement		
Involved Not involved	Reference 0.136 (0.047–0.399)	0.0002
Comorbidity		
Ongoing DM	Reference	
Denovo DM CKD AML Kidney transplantation COVID-19 + Systemic steroid	0.583 (0.232–1.464) 0.609 (0.080–4.659) 6.809 (1.821–25.461) 6.276 (0.767–51.344) 0.000 (0.000)	0.251 0.633 0.004 0.087 0.980
COVID-19 infection		
Present Absent	Reference 0.797 (0.352–1.807)	0.587

**Table 5:** Multivariate Cox regression analysis of the prognostic factors associated with survival

Variable	HR (95% CI)	*P*-value
Age	1.184 (1.095–1.281)	< 0.0001
Stage	6.848 (1.326–35.374)	0.022
Number of surgical interventions	0.322 (0.083–1.248)	0.101
Gender		
Male Female	Reference 0.453 (0.152–1.345)	0.154
Orbital involvement		
Involved Not involved	Reference 1.123 (0.129–9.763)	0.917
Comorbidity		
Ongoing DM Denovo DM CKD AML Kidney transplantation COVID-19 + Systemic steroid	Reference 0.335 (0.105–1.066) 14.146(0.956–209.413) 1.987 (0.391–10.101) 318.095 (8.209–12,326.636) 0.000(0.000)	0.064 0.054 0.408 0.002 0.985
COVID-19 infection		
Present Absent	Reference 1.062 (0.311–3.622)	0.924

## Discussion

**Fig. 2:**
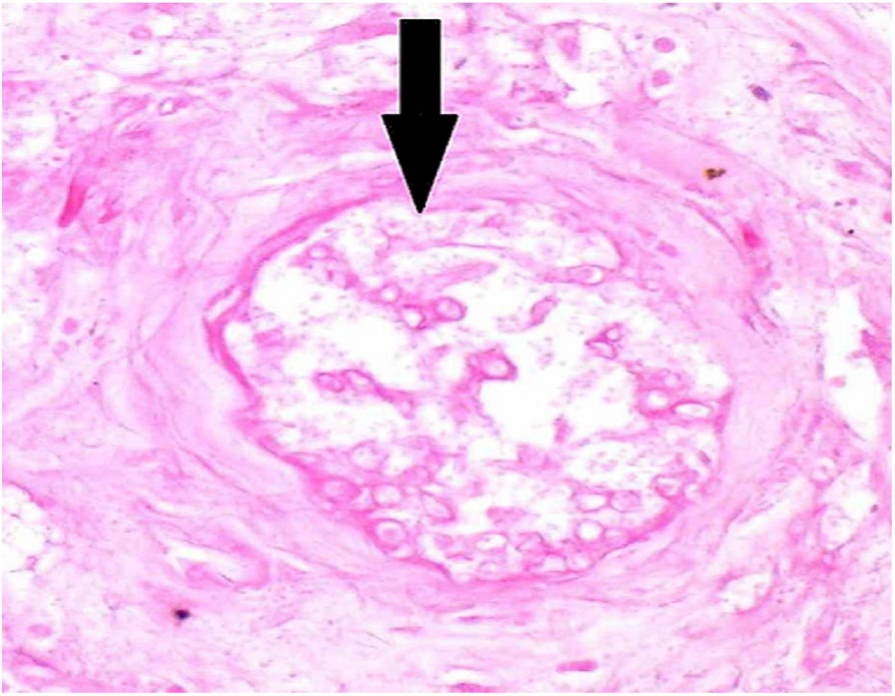
Histological examination with hematoxylin and eosin stain shows angioinvasion by broad, aseptate hyphae with right angle branching, corresponds with Mucor species

**Fig. 3:**
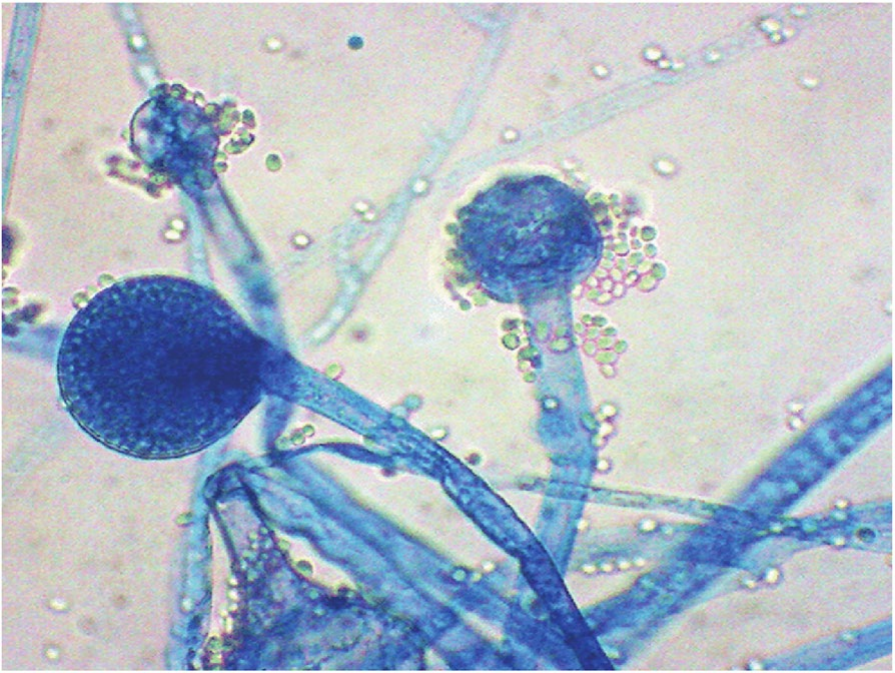
Mucor species-microscopic morphology in culture, Lactophenol cotton blue staining shows broad aseptate hyphae and a sporangium with aggregation of sporangiospores

**Fig. 4:**
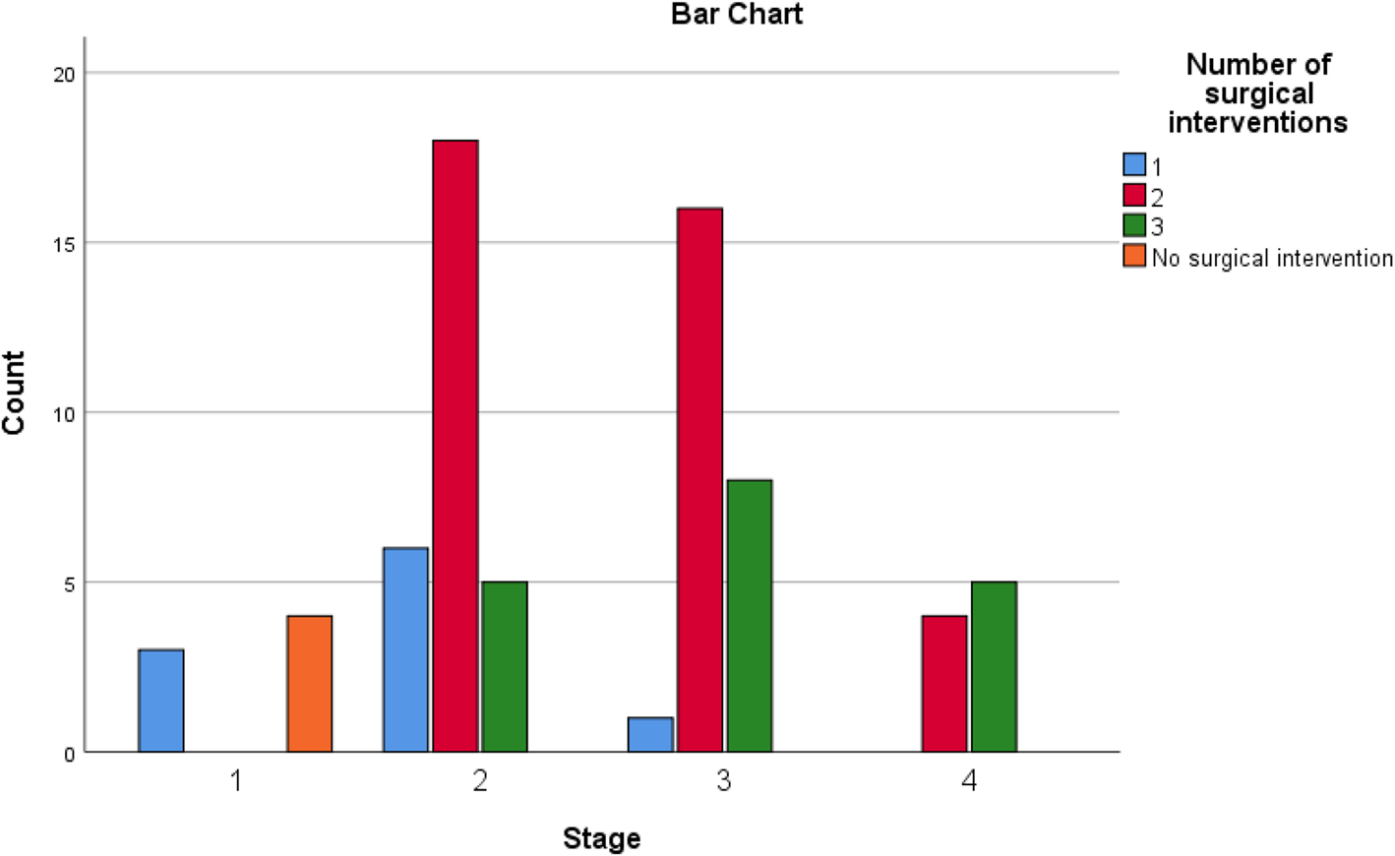
The number of surgeries needed according to the stage of AIFRS

**Fig. 5:**
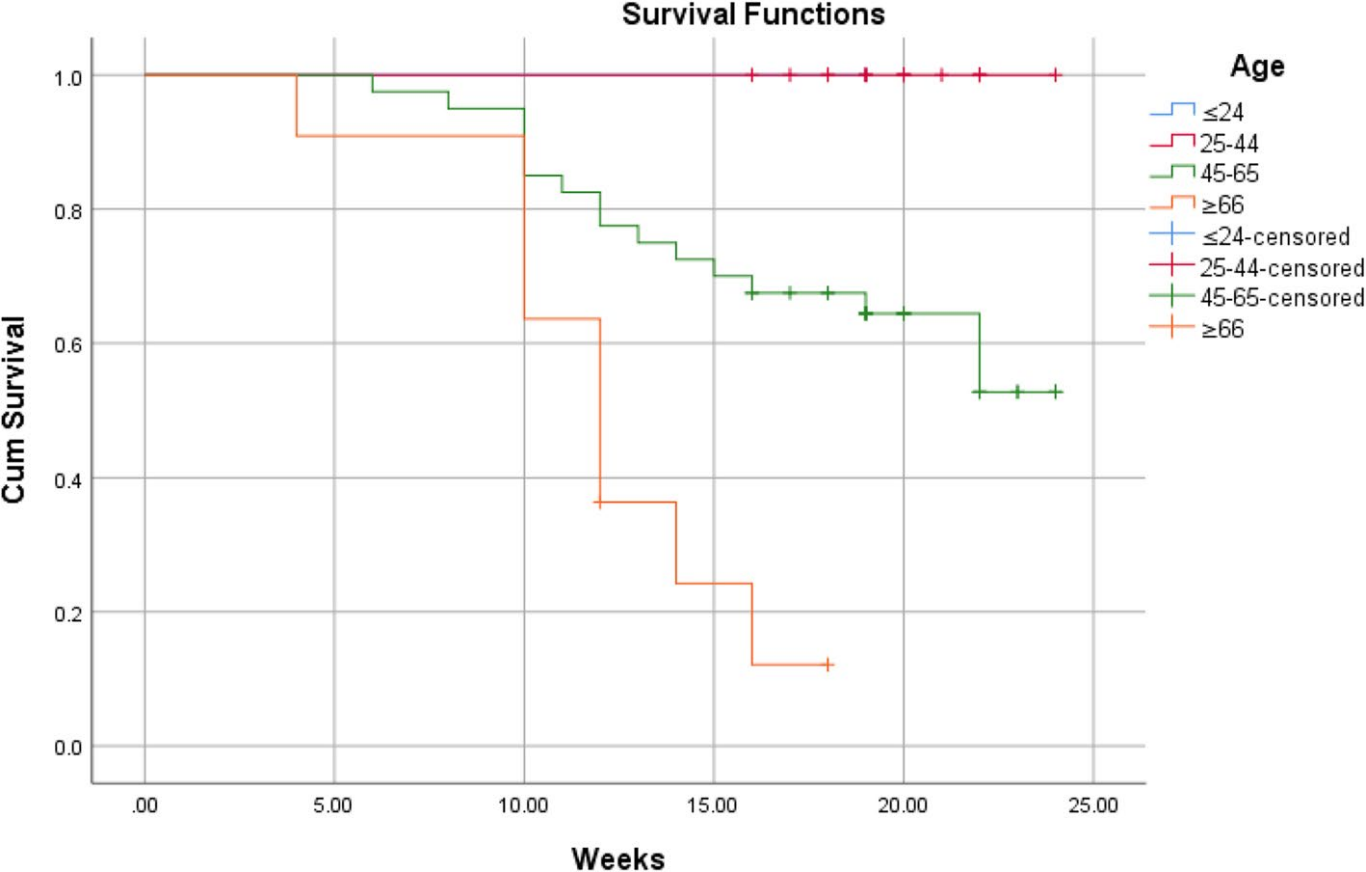
Kaplan–Meier survival curves by age

**Fig. 6:**
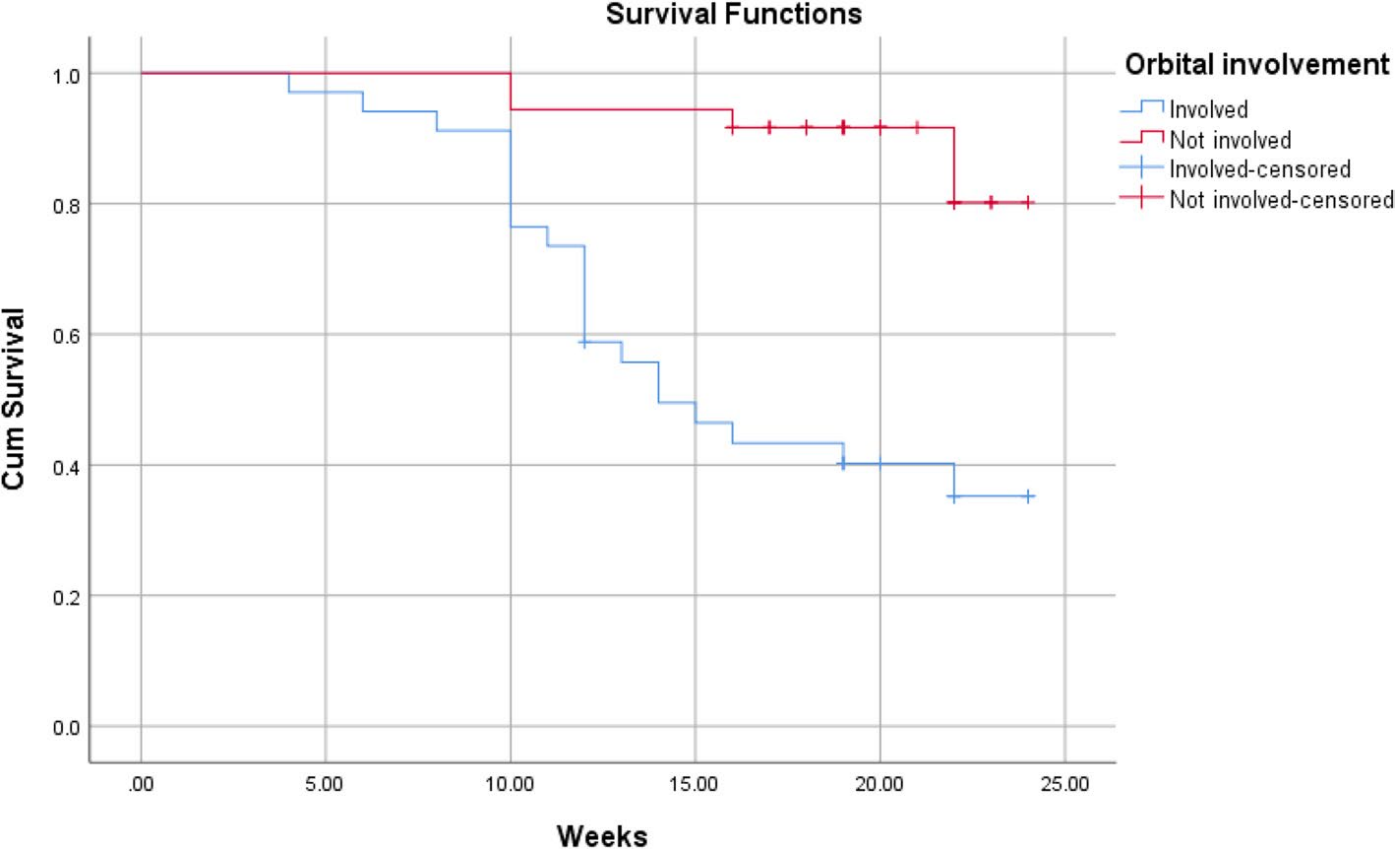
Kaplan–Meier survival curves by orbital involvement

**Fig. 7:**
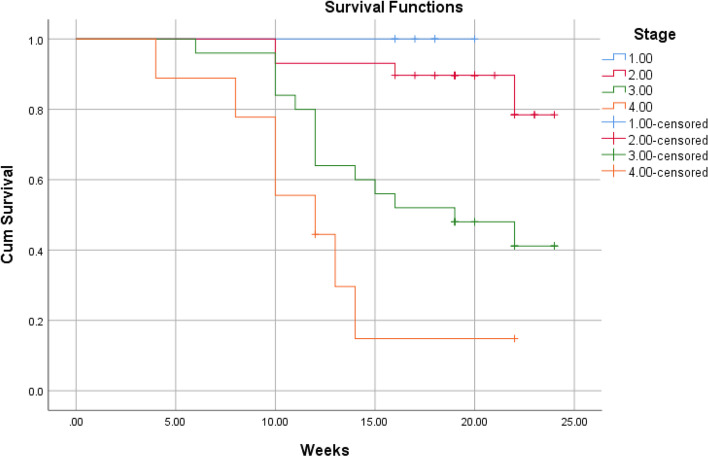
Kaplan–Meier survival curves by staging

**Fig. 8:**
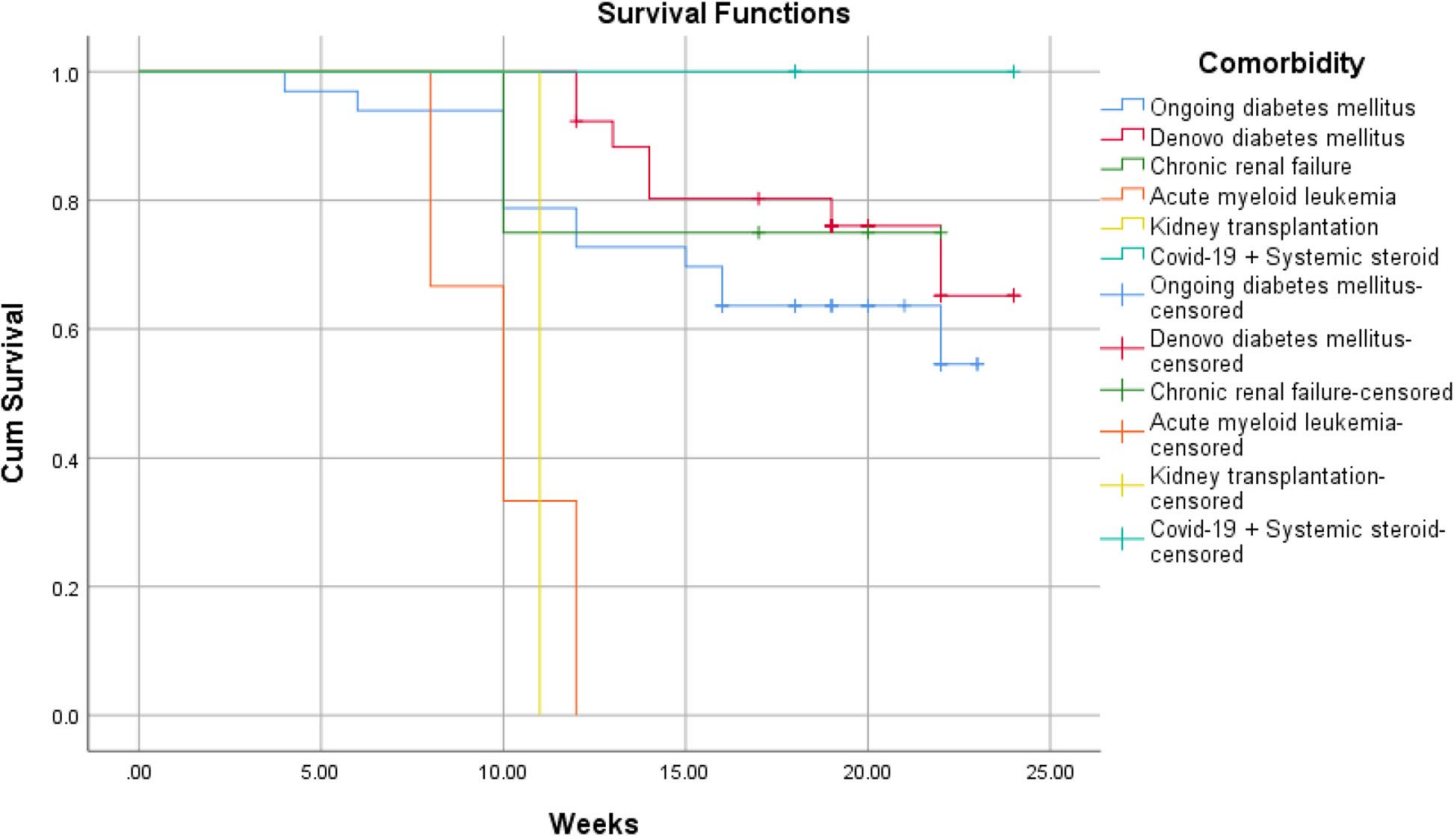
Kaplan–Meier survival curves by comorbidity

The aim of this study is to determine the outcomes of management of AIFRS in our tertiary medical center and the factors that are associated with the prognosis. We reported 70 cases of AIFRS during the period between January 2021 and July 2022. The mean age of patients was 52.5 years. A decrease in mean age of AIFRS cases was noted in our study compared with a retrospective review, which was performed in a tertiary academic medical center of patients with AIFRS from January 2009 through February 2019, and showed higher average of age (57.3 years) [4]. This decrease in ages of patients of AIFRS could be due to the increasing number of young patients who were rarely infected with this disease before COVID-19 pandemic. Pathognomonic signs and symptoms of AIFRS have not been determined, and the presenting symptoms are often nonspecific. However, facial edema, facial pain, fever, and nasal obstruction have been reported most commonly [22, 23]. Regarding to our study, facial pain and swelling, followed by headache were the most common presenting complaint. Persistent nasal complaints of immunocompromised patients must prompt doctors to obtain radiological investigations and nasal endoscopy in order to determine the presence and the stage of AIFRS, then the proper plan for management. Actually, there is no consensus staging system for AIFRS. Establishing a disease staging system may help speed up and guide treatment methods which lead to improve the outcomes and prognosis of disease. We used a simple staging system derived from a previous study which used more detailed system [24]. Radiological assessment by using CT and/or MRI can help in diagnosis of AIFRS as well as develop the appropriate treatment plan. The most common CT findings of AIFRS is unilateral mucosal thickening of the nasal cavity [4]. In the present study, we found more advanced disease since the most commonly reported radiological findings were sinus mucosal disease (stage 2) followed by orbit involving disease (stage 3). There were a few cases that had an intracranial extension (stage 4) and less cases had limited mucosal disease of nasal cavity (stage 1). The vast majority of cases were unilateral, while bilateral infection was rare (2.9%). Although histological examination is necessary to confirm the diagnosis (Fig. 2), direct microscopic examination and fungal culture can help in differentiation between different types of fungi, since each fungus has its own specific hyphae [25, 26]. Mucor has broad, large aseptated hyphae with right-angle branching (Fig. 3), while Aspergillus presents septated hyphae with branching at 45° angles [25, 26]. However, in the current study, the diagnosis of AIFRS was established depending on histological examination for all patients, while direct microscopic examination and/or fungal culture were used to determine the causative fungi. The literature is confounded among publications regarding to causative organisms. Some studies identified Aspergillus as the most common organism, while others considered Mucor more common. Craig and colleagues found in a review that the most common isolated fungus was Mucor [1]. In contrast, several other studies revealed Aspergillus as the most common causative fungus of AIFRS [16, 27, 28]. During the recent COVID-19 pandemic, Mucor species became the most common organisms causing AIFRS [2]. Regarding to our study, the vast majority of cases were caused by Mucor, while Aspergillus was the causative organism in limited number of cases. Two studies showed that Mucor species can be more aggressive and more invasive to neurovascular and orbital structures, leading to poor outcomes [20, 29]. Other studies suggested that the type of fungus does not affect the prognosis [11, 30, 31]. Mucor and Aspergillus co-infections are rare entities. Few cases have been reported in the literature with favorable clinical outcomes [32]. Tabarsi et al. reported two cases of AIFRS with Mucor and Aspergillus co-infection and the two patients survived without recurrence on follow-up [32]. Of our cases, three of them showed co-infection with Mucor and Aspergillus. All of them survived without recurrence on three months of follow-up. However, there was no significant correlation between the type of causative fungus and the outcome (*P* = 0.2). Many western studies found that haematological malignancies are the most common risk factors for AIFRS [33]. However, diabetes mellitus was found to be the main cause of AIFRS during COVID-19 pandemic particularly in low and middle-income countries where control diabetes could be challengeable [21, 34–37]. This is consistent with our study as DM, whether ongoing or denovo, was found to be the main comorbidity that associated with AIFRS. The prevalence of AIFRS increased dramatically during the COVID-19 era. In 2020, the World Health Organization (WHO) recommended the use of corticosteroids in management of severe cases of COVID-19 disease who required supplemental oxygen and mechanical ventilation, while no benefits of corticosteroids were noticed in patients who did not require supplemental oxygen during the course of illness [38]. Unfortunately, the indiscriminate and uncontrolled use of corticosteroids in management of COVID-19 patients lead to an upsurge of AIFRS cases particularly in developing countries [32, 39–42]. Several factors have been suggested to be the cause of AIFRS in patients with active COVID-19 infection including high glucose (diabetes, new onset hyperglycemia, steroid-induced hyperglycemia), low oxygen (hypoxia), high iron levels (increased ferritins), decreased phagocytic activity of white blood cells due to immunosuppression (SARS-CoV-2 mediated or steroid-mediated), acidic medium (metabolic acidosis, diabetic ketoacidosis), and prolonged hospitalization with or without mechanical ventilation [2]. In our study, however, 33 cases (47.1%) had previous history of COVID-19 infection with systemic steroid therapy. Early diagnosis and management of AIFRS is crucial for better results. A summary of findings of studies of AIFRS among COVID-19 patients is shown in Table 6. Unfortunately, patients with AIFRS frequently present with nonspecific symptoms including nasal congestion, fever, facial pain, and others, leading to delay of correct diagnosis and management [4]. In our study, we found that the most common presenting symptoms were facial pain and swelling followed by headache. Physicians must have very high clinical suspicion of AIFRS if COVID-19 patients exhibit any signs or symptoms listed in Table 7 [24]. Chamilos et al. showed in his study that a delay of treatment ≥ 6 days can lead in some patients to twofold increase in mortality rate during 12 weeks of follow-up [43]. Yohai et al. studied 145 cases of AIFRS and found that the delay of antifungal treatment more than 6 days has more profound effect on survival rate than delay of surgery [44]. In contrast, other recent study revealed no statistically significant difference in survival rate between cases which underwent surgery during 1–30 days after diagnosis [31]. Interestingly, we found that the longer period between the onset of symptoms and the commencement of the management, the better the survival (*P* ≤ 0.0001) (Table 3). Wandell and colleagues suggested that this could be explained by a more indolent disease process in this group [27]. additionally, having orbital or neurological symptoms in advanced cases prompt the patient for medical consultation quickly. Multidisciplinary approach should be applied with focus on antifungal therapy, extensive surgical debridement, and reverse the underlying immunodeficiency causes. In 2019, the ECMM in cooperation with the MSG ERC, developed a comprehensive guidance to help in clinical decision-making regarding to mucormycosis. The guidelines strongly recommended complete surgical debridement as fast as possible, in addition to systemic antifungal treatment [21]. Surgical debridement should be aggressive to remove all the necrotic tissues until bleeding is apparent. This result can be achieved by TESS in most cases, but sometimes combined approaches are necessary depending on extent of the disease. In our study, we used external approach in addition to TESS in management of 4 patients due to extensive disease. Debridement should be repeated as required. Our results revealed that over half cases required two surgical interventions, while limited number did not need surgical debridement. The vast majority of patients of stage 2 and stage 3 needed two surgical debridement, while all patients of stage 1 who were treated surgically needed just one. The Bar chart in Fig. 4 shows the number of surgeries which were needed relative to the stage. Actually, clear recommendations about which and when patients should undergo orbital exenteration have not determined by previous studies. Turner et al. studied 80 patients who underwent orbital exenteration and he found that survival rate did not improve [11]. Similar result was found by Hargrove and colleagues in his meta-analysis of 224 patients [45]. However, several factors should be taken into consideration while thinking of performing orbital exenteration such as orbital and intracranial extension and overall prognosis [1]. Vengerovich and colleagues chose to perform orbital exenteration in cases where the disease had good prognosis and the surgeon thought that he can remove all the infected tissues by exenterating the orbit, but if the disease was very advanced with bilateral orbital or intracranial involvement and the prognosis was poor, then he did not conduct orbital exenteration [4]. Several other studies showed similar opinions [1, 11, 30, 31]. In the present study, we performed orbital exenteration in limited cases when patient had good prognosis with extreme orbital involvement without any vision or eye movement. Amphotericin B deoxycholate has been used as the drug of choice for decades. Although it is effective, it has substantial toxicity especially in high doses and long periods of treatment. Therefore, its use should be preserved for sittings in where other antifungal agents are not available [21, 46, 47]. According to the ECMM and MSG ERC guidelines, liposomal amphotericin B is the first-line antifungal therapy for mucormycosis, and the minimum recommended dose of liposomal amphotericin B is 5 mg/kg per day. The dose should be raised to 10 mg/kg per day for progressive disease or if brain involvement or solid organ transplantation was present [21]. Daily doses reported by other studies ranged from 1 to 10 mg/kg per day [48, 49]. Increased doses until 10 mg/kg per day were associated with increased response rates [49], while doses more than 10 mg/kg per day did not result in higher response rates. doses below 5 mg/kg per day are recommended with marginal strength only. Posaconazole and isavuconazole are considered as the second line agent when amphotericin B lipid formulations are not available [21]. The duration of treatment of AIFRS is undetermined through the medical literature. In general, intravenous therapy should be continued until signs and symptoms of AIFRS disappear, full radiological improvement is noticed, and immunodeficiency status is permanently reversed. Using of oral isavuconazole or posaconazole as a maintenance therapy is strongly recommended [21]. Azole antifungal agents were demonstrated to be more effective than amphotericin B in treating AIFRS caused by Aspergillus with voriconazole being considered the first-line antifungal choice [1]. Recently, two other agents from second generation azole drugs have been reported to be effective against aspergillosis and mucormycosis, posaconazole and isavuconazole, and they are less hepatotoxic than voriconazole and less nephrotoxic than amphotericin [1, 21]. Regarding to our study and as a result of high price of liposomal amphotericin B and the inability of patients to obtain it, the used doses ranged between 2–3 mg/kg per day which is lower than the recommended doses. In fact, this dosage was revealed to be effective in patients with younger ages (≤ 44 years), early stages (1 and 2), new-onset comorbidities (denovo DM, COVID-19 infection, short-term systemic steroid therapy), and orbit sparing disease, since the survival rate in these groups was high (Table 3). Existing literature reveals unclear results regarding the benefit of using a combination of amphotericin B with azole antifungals although some studies (mostly case reports) showed positive results [50, 51]. Due to the lack of strong evidence, it is difficult to advise using a particular combination of antifungals. However, when amphotericin B monotherapy is inadequate, especially when suboptimal dosage are used, a combination therapy of amphotericin B and azole agents may be considered. The mortality of AIFRS is high, ranging from 40–80%. There is controversy in medical literature about the importance of extent of the disease on outcome. Monroe and colleagues found that orbital or intracranial extension associated with worse outcome [30]. The same result was found by Cornely and colleagues [21]. while Wandell and colleagues revealed that extent of the disease does not affect the prognosis [27]. Two theories have been suggested to interpret the poorer outcome in patients with orbital involvement disease. The first one is that when the fungus reach the orbit it can easily access the intracranial space through the ophthalmic artery, superior orbital fissure, or optic canal [52]. The second theory is that the fungus infections that can spread out the paranasal sinuses into surrounding structures could be more inherently virulent [44]. The treatment with amphotericin B may reduce the mortality from 92 to 41%. Combined therapy with amphotericin B and surgical debridement can improve survival by 15–30% compared to medical management alone [53, 54]. Pandey and colleagues reported a mortality rate of 21% in his study [54]. Similarly, Rao and colleagues found that the mortality rate was around 16.1% [55]. Regarding to our study, the overall 3-month mortality rate was about 35.7%, which is higher than aforementioned studies. More specific, the mortality rate for patients of stage 3 and stage 4 was extremely higher (77.8% and 61.8%, respectively). Many studies subscribed several negative prognostic factors such as delayed diagnosis, diabetes, advanced age, orbital or intracranial involvement, and Mucor species [11, 20, 30, 33, 44, 45, 56, 57]. This is consistent with our results, since younger ages had higher survival rate. All patients younger than 44 years survived, while near half of patients over 44 years died (Fig. 5). Significant decline in survival rate was noticed for orbit involving cases (Fig. 6). Similarly, the survival rates for patients in stage 3 and stage 4 were less than patients in stage 1 and stage 2 (Fig. 7). Regarding to comorbidity, we found that the patients with AML and kidney transplantation had the highest mortality rate, while patients with denovo DM and those with COVID-19 infection and systemic steroid therapy had the best prognosis (Fig. 8).

**Table 6:** Summary of findings of studies of AIFRS among COVID-19 patients

Reference	Study design	Age (mean ± SD) (years)	Underlying diseases	Presenting symptoms and signs	Risk factors	Causative organism	Antifungal treatment	Mortality rate (%)
El‐Kholy et al. (2020), [10]	Longitudinal prospective study, tertiary referral center	52.92 ± 11.30	DM, malignancy, HTN, CKD, asthma, CAD, Hypothyroidism, SLE	Headache, facial numbness	Systemic corticosteroid therapy for COVID-19	Mucor, Aspergillus	Amphotericin B, voriconazole, posaconazole	36.11
Tadros et al. (2020–2021), [58]	Longitudinal prospective study, tertiary referral center	57.17 ± 14.7	DM, CKD, malignancy, HTN	Headache and facial pain, orbital pain, preorbital edema	Systemic corticosteroid therapy for COVID-19	Mucor, Aspergillu, mixed fungal infections	Amphotericin B, voriconazole, posaconazole	33.33
Roushdy et al. (2021), [59]	Case series, tertiary referral center	67.75 ± 10.5	DM, CKD, HTN, malignancy, CAD	Facial swelling, complete ophthalmoplegia, proptosis, ptosis, hard palate, sluggish ulcer	Systemic corticosteroid therapy for COVID-19	Mucor	Amphotericin B	25
Pakdel et al. (2021), [60]	Cross-sectional descriptive multicenter study	Median 52 years (range 14–71)	DM, HTN, malignancy, asthma, CAD, hepatic cirrhosis, hypothyroidism, tuberculosis	Facial swelling, ptosis, proptosis, acute vision loss, cranial nerve palsies, otological symptoms	Systemic corticosteroid therapy for COVID-19, DKA	Mucor	Amphotericin B, posaconazole, caspofungin, combined therapy	47
Eldsouky et al. (2020) [61]	Cross‐sectional cohort study	59.6 ± 11.9	Chest disease, CAD, DM, HTN, malignancy	Headache and facial pain, ophthalmoplegia, visual loss, and blindness	Antibiotic, corticosteroid, oxygen therapy	Mucor, Aspergillus	Amphotericin B	13.3

*HTN* hypertension, *CAD* coronary artery disease, *SLE* systemic lupus erythematosus, *DKA* diabetes ketoacidosis

**Table 7:** Warning symptoms and signs of AIFRS among COVID-19 patients

• Nasal discharge—mucoid, purulent, blood-tinged or black
• Nasal mucosal erythema, inflammation, purple or blue discoloration, white ulcer, ischemia, or eschar
• Nasal stuffiness
• Epistaxis
• Foul smell
• Worsening headache
• Facial pain
• Regional pain – orbit, paranasal sinus or dental pain
• Ocular motility restriction, diplopia
• Eyelid, periocular or facial edema
• Eyelid, periocular, facial discoloration
• Sudden ptosis
• Proptosis
• Sudden loss of vision
• Fever, altered sensorium, paralysis, focal seizures
• Facial paresthesia, anesthesia
• Facial palsy

## Conclusion

AIFRS is a devastating disease with high mortality rate. Early diagnosis and immediate medical and surgical management could improve the survival rate. Even if surgical debridement is delayed due to patient's general condition, antifungal treatment should be initiated immediately. Low doses of liposomal amphotericin B are less effective in cases with poor prognosis (older ages, involved orbits, advanced stages, chronic immunodeficiency, haematologic malignancies, and organs transplantation) and high doses are highly recommended. However, more studies are required to strengthen such results.

### Strengths and limitations

Several limitations were found to our study. The sample size was small, therefore, the value of Cox regression analysis results is limited and more reliable results could be obtained by larger sample. A longer follow up should be applied to get better perception about survival and risk factors. We did not perform fungal culture routinely, due to limited resources, which is strongly recommended for identification of species and for antifungal susceptibility testing.

## Data Availability

The corresponding author will provide the data supporting this research’s findings upon a reasonable request.

## Abbreviations

AIFRS: Acute invasive fungal rhinosinusitis
CT: Computed tomography
MRI: Magnetic resonance imaging
SD: Standard deviation
CNS: Central nervous system
DM: Diabetes mellitus
AML: Acute myeloid leukemia
CKD: Chronic kidney disease
HTN: Hypertension
CAD: Coronary artery disease
SLE: Systemic lupus erythematosus
DKA: Diabetes ketoacidosis
